# Microsporum canis-Induced Tinea Capitis: A Rapid Screening Algorithm

**DOI:** 10.7759/cureus.55919

**Published:** 2024-03-10

**Authors:** Jesús Iván Martínez-Ortega, Samantha Franco González, Brayant Martinez-Jaramillo, Arely Gissell Ramirez Cibrian

**Affiliations:** 1 Dermatology, Instituto Dermatológico de Jalisco "Dr. José Barba Rubio", Zapopan, MEX; 2 Internal Medicine, Centro Médico Nacional Siglo XXI, Ciudad de México, MEX; 3 Medical Benefits, Mexican Institute of Social Security, Campeche, MEX

**Keywords:** alopecia, dermatophyte scalp infection, microsporum, trichophyton, tinea capitis

## Abstract

Tinea capitis is a common fungal infection of the scalp, primarily affecting children, and caused by fungi like *Trichophyton* and *Microsporum*. Its pathogenesis is influenced by both host-specific and environmental factors, resulting in various clinical presentations including hair loss and scaling of the scalp. We present the case of an eight-year-old male with tinea capitis, characterized by itching and hair loss in the occipital area. Examination revealed characteristic findings on trichoscopy, and direct examination of hair confirmed parasitization. Treatment with terbinafine was initiated, leading to the resolution of symptoms. Epidemiological variations in the etiology of tinea capitis exist globally, with *Trichophyton* predominating in some regions and *Microsporum *in others. Trichoscopy is a valuable diagnostic tool for differentiating fungal infections, guiding treatment decisions. Despite the efficiency of direct skin and hair examination, the common occurrence of tinea and the lack of mycological centers in many clinics pose challenges. To address this, we propose integrating trichoscopy and epidemiological and clinical data for a quick in-office decision tool.

## Introduction

Tinea capitis, a dermatophyte scalp infection, is predominantly observed among the pediatric population and is caused by two primary fungi species: *Trichophyton* and *Microsporum*. The pathogenesis of tinea capitis is influenced by the microorganism itself, host-specific variables, and environmental factors, leading to clinical presentations such as subtle hair loss with scalp scaling, alopecia featuring scaly patches, or alopecia with black dots, particularly prevalent in pediatric cases [1,2].

## Case presentation

**Figure 1:**
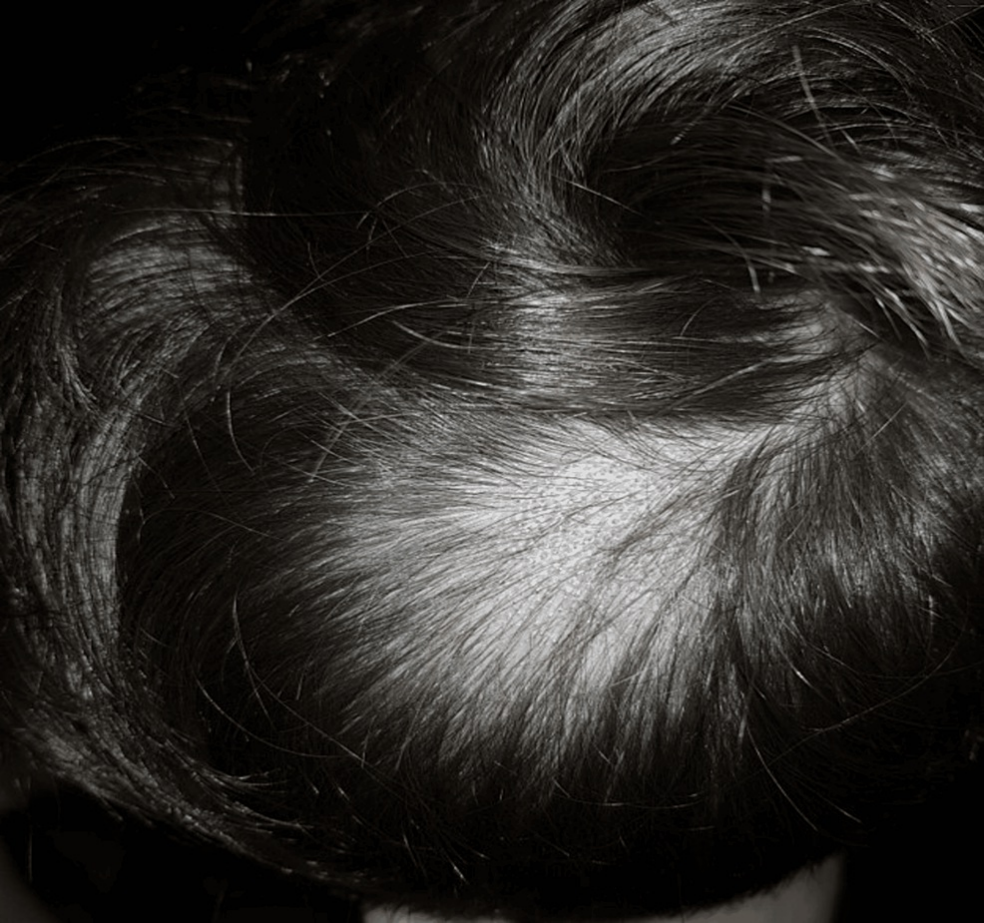
Clinical picture of the lesion on the head.

**Figure 2:**
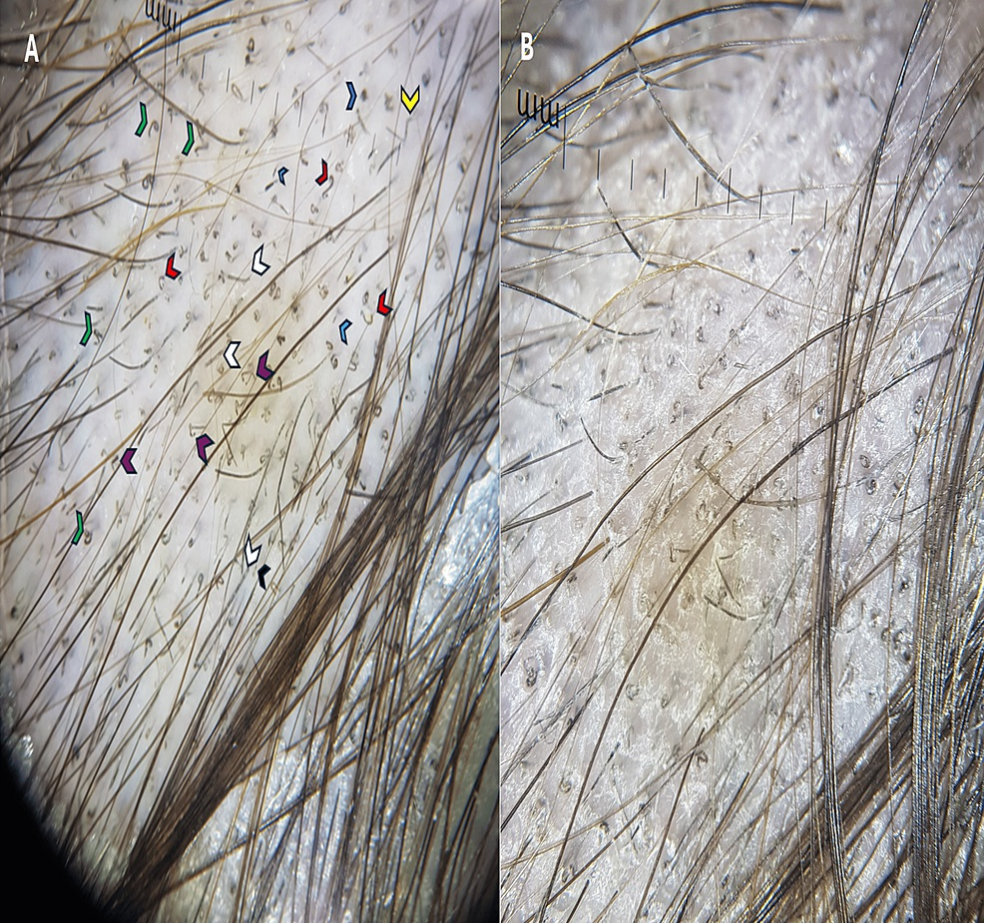
Trichoscopy. Trichoscopy image of the lesion with gel on the left panel (A), featuring comma hairs (red arrowheads), Morse code-like hairs (white arrowheads), black dots (blue arrowhead), corkscrew hairs (green arrowhead), broken dystrophic hairs (blue light arrowhead), block hair (black arrowhead), zigzag hair (yellow arrowhead), and bent hair (purple arrowhead). The right panel (B), without gel, shows perifollicular and diffuse scaling.

An eight-year-old male patient from Yucatan presented with a one-week history of itching and hair loss in the occipital area. Upon examination, an alopecia scaly patch, measuring 4 cm in diameter, was observed. Trichoscopy revealed numerous comma hairs, corkscrew hairs, Morse code-like hairs, and other findings (Figure 1). Direct examination of the hair indicated parasitization of the ectoendothrix type (Figure 2). Notably, the patient had not used topical steroids, and there was no exposure to pet houses or animals.

**Figure 3:**
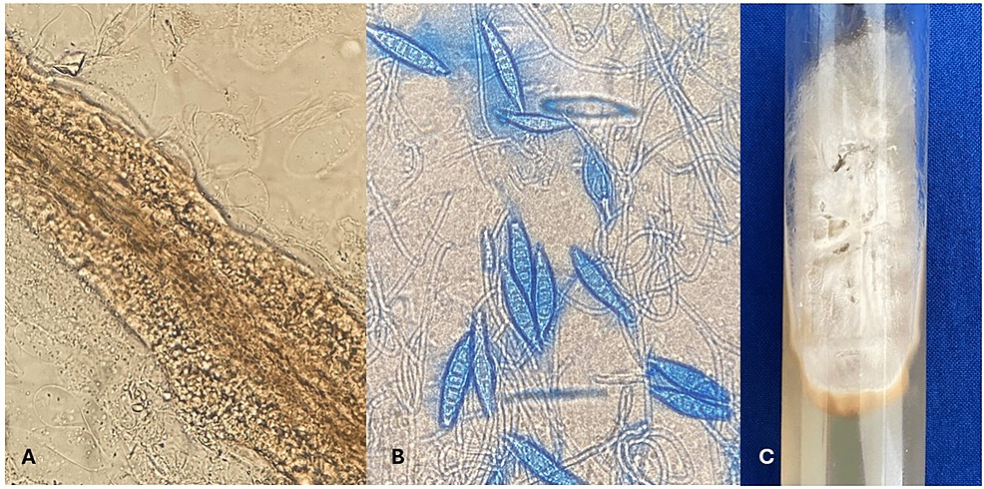
Morphological characteristics and culture. Panel A (left) illustrates the direct examination of the hair with 20% KOH (20x), indicating parasitization of ectoendothrix type. The micromorphology of the culture in lactophenol blue (20x), as depicted in panel B, reveals abundant macroconidia with thick-walled and pointed ends, exhibiting more than six septa inside, along with hyaline hyphae and scarce microconidia. Panel C describes the macroscopic characteristics of the colony on Sabouraud agar at 12 days, presenting as a powdery white mold. KOH: potassium hydroxide

Based on this data, we integrated the diagnosis of tinea capitis, and terbinafine 125 mg was administered orally daily for one month. After two weeks, the culture exhibited a powdery white mold aspect (Figure 3, panel C). The micromorphology of the culture in lactophenol blue revealed abundant macroconidia with thick-walled and pointed ends, more than six septa inside, along with hyaline hyphae and scarce microconidia (Figure 3, panel B), corresponding to *Microsporum canis*.

Regrettably, although the patient reported complete recovery over the phone after one month with no symptoms and apparently no signs by the description, they did not attend the follow-up appointment.

## Discussion

Considering the epidemiological landscape, the etiology of tinea capitis varies across regions. In numerous African, American, and Asian countries, *Trichophyton* is the leading cause among children, while in many European nations, *Microsporum* takes precedence [3]. In Mexico, characterized by geographic and ethnic diversity, preliminary data indicates regional variations, with the north region showing a higher prevalence of *Trichophyton* and the western and central regions favoring *Microsporum* [4]. There is limited data available for the southern region; preliminary results from a center situated in Yucatan, with a data span of 22 years, detected *Microsporum *spp. in 146 (36%) cases out of 479.

Trichoscopy, initially introduced in 2008 for the evaluation of tinea capitis [5], has evolved into a valuable tool for diagnosis and management in this context. It serves as a differential diagnostic tool for various alopecia etiologies, aids in distinguishing between *Microsporum* and *Trichophyton* infections, and facilitates the monitoring of treatment response [6].

A recent systematic review with 536 patients emphasized the high specificity and positive predictive value of trichoscopy features in tinea capitis, including comma hairs (99%/94%), corkscrew hairs (100%/98%), and Morse code-like hairs (100%/100%) [7]. While the sensitivity is comparatively modest, combining trichoscopy signs with clinical features and epidemiological data has the potential to augment sensitivity, transforming it into a plausible screening tool. Notably, no existing studies are measuring these three variables in conjunction.

Trichoscopy also proves useful in distinguishing between *Microsporum* and *Trichophyton* infections, with specific patterns such as Morse code-like hairs, zigzag hairs, bent hairs, and diffuse scaling being indicative (only present) of *Microsporum* tinea capitis [7]. This differentiation is crucial as treatment strategies differ, with griseofulvin being more effective for *Microsporum*, while terbinafine or itraconazole is preferred for *Trichophyton *[6]. Regrettably, griseofulvin was unavailable in our setting, leading us to choose terbinafine as an alternative.

**Figure 4:**
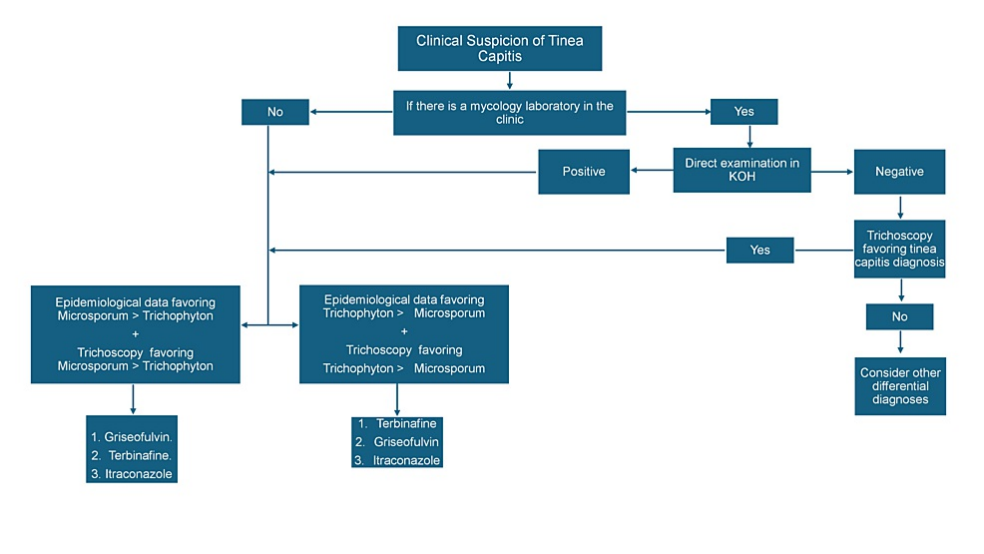
Tinea capitis proposed protocol. KOH: potassium hydroxide

In contrast to the gradual resolution of perifollicular and diffuse scaling, the disappearance of dystrophic hairs, such as comma hairs, corkscrew hairs, zigzag hairs, Morse code-like hairs, broken hairs, and black dots, typically occurs relatively soon, within 4-12 weeks after initiating therapy [7] (Figure 4).

## Conclusions

While culture remains the gold standard for diagnosing dermatophyte infections, it typically takes at least two weeks. Direct examination is a useful, easy, and rapid tool; however, many clinics and hospitals in undeveloped countries lack mycological laboratories. In contrast, trichoscopy is emerging as an increasingly valuable and versatile tool in tinea capitis. To address the challenge of timely diagnosis, we propose an algorithm that incorporates trichoscopy along with clinical and epidemiological data as a rapid screening tool. This approach is designed to assist first-contact clinicians in early suspicion, prompt and optimal treatment selection, and effective follow-up, ultimately contributing to improved patient care.
